# Amorphous Tin Oxide Applied to Solution Processed Thin-Film Transistors

**DOI:** 10.3390/ma12203341

**Published:** 2019-10-14

**Authors:** Christophe Avis, YounGoo Kim, Jin Jang

**Affiliations:** Advanced Display Research Center and Department of Information Display, Kyung Hee University, Seoul 130-701, Korea; ygkim@tft.khu.ac.kr (Y.K.); jjang@khu.ac.kr (J.J.)

**Keywords:** tin oxide, thin-film transistor, hafnium oxide, solution process, amorphous oxide semiconductor

## Abstract

The limited choice of materials for large area electronics limits the expansion of applications. Polycrystalline silicon (poly-Si) and indium gallium zinc oxide (IGZO) lead to thin-film transistors (TFTs) with high field-effect mobilities (>10 cm^2^/Vs) and high current ON/OFF ratios (I_On_/I_Off_ > ~10^7^). But they both require vacuum processing that needs high investments and maintenance costs. Also, IGZO is prone to the scarcity and price of Ga and In. Other oxide semiconductors require the use of at least two cations (commonly chosen among Ga, Sn, Zn, and In) in order to obtain the amorphous phase. To solve these problems, we demonstrated an amorphous oxide material made using one earth-abundant metal: amorphous tin oxide (a-SnO_x_). Through XPS, AFM, optical analysis, and Hall effect, we determined that a-SnO_x_ is a transparent n-type oxide semiconductor, where the SnO_2_ phase is predominant over the SnO phase. Used as the active material in TFTs having a bottom-gate, top-contact structure, a high field-effect mobility of ~100 cm^2^/Vs and an I_On_/I_Off_ ratio of ~10^8^ were achieved. The stability under 1 h of negative positive gate bias stress revealed a Vth shift smaller than 1 V.

## 1. Introduction

Amorphous oxide semiconductors (AOS) with high mobility ~10 cm^2^/Vs and high transparency (>80% in the visible region) [1] are under extensive research and are considered for wide application in electronics, such as displays [2]. In addition, AOS-based thin-film transistors (TFTs) provide new manufacturing possibilities for applications, such as flexible devices [3].

Amorphous oxide semiconductors use multiple post-transition metal cations, usually chosen among indium, zinc, tin, and gallium. Being fabricated by vacuum process or solution process, indium gallium zinc oxide (IGZO), zinc tin oxide (ZTO), indium zinc tin oxide (IZTO), and indium zinc oxide (IZO) have been investigated and have shown the potential for careful selection of cations and processes for high-performance AOS TFTs [4,5,6]. High-k dielectrics (Al_2_O_3_, HfO_2_, ZrO_2_, Y_2_O_3_, etc.) have also improved the performances of oxide TFTs [7,8,9,10,11].

On the other hand, single cation metal oxide materials (ZnO, In_2_O_3_, SnO_2_) demonstrate a polycrystalline phase. For example, In_2_O_3_ leads to high-performance TFTs but doping is usually required to obtain high-performance and stable TFTs [5,12]. The amorphous phase is preferred for its higher uniformity over large areas for electronics applications.

Tin oxide, SnO_2_, was the first transparent conducting oxide (TCO) material used as a channel region in a TFT [13]. Since then, because SnO_2_ is an n-type semiconductor and SnO a p-type semiconductor, extensive research has been conducted for large area electronics [14,15] and its potential use in complementary metal oxide semiconductors (CMOS). Nevertheless, one of the major issues has been the small I_on_/I_off_ ratio due to the high conductivity of polycrystalline SnO_2_ [14,16].

Recently, solution processed polycrystalline SnO_2_ TFTs have regained interest because of their high field-effect mobility of 40, and even 90 cm^2^/V has been achieved, but the devices lacked either high I_on_/I_off_ ratios or low leakage currents [17,18]. Although the amorphous phase of tin oxide made via solution process was reported, it was not applied as the active material in TFTs [17]. Vacuum processed amorphous tin oxide used in TFTs has been reported, but the field-effect mobility was lower than 1 cm^2^/Vs [19]. On the other hand, vacuum processed polycrystalline SnO_2_ was used in TFTs leading to mobilities of 147 cm^2^/Vs [20].

In this work, we developed amorphous tin oxide (a-SnO_x_) using a solution process and investigated the optical (i.e., transmittance, optical bandgap), physical (i.e., surface roughness, crystallinity), chemical, and electronic (i.e., composition, phases, nature of bonds) properties. The material was then used as the active material in TFTs, which had HfO_2_ as the dielectric. The TFTs showed exceptional high mobility (~100 cm^2^/Vs) and high I_on_/I_off_ ratios (~10^8^), and low leakage currents. Justified by the TFTs’ remarkable high performances, various stresses such as positive bias stress (PBS) and negative bias stress (NBS) were applied to evaluate the practical use of the TFTs in large area applications. This work is innovative in that it demonstrates the development and usage of an amorphous oxide material having only one metal, and shows stable TFTs competing with vacuum processed counterparts for large area electronics.

## 2. Materials and Methods 

### 2.1. Precursor Solutions

The SnO_x_ and HfO_2_ precursor solutions were made by mixing SnCl_2_ (99.99%, metals basis) or HfCl_4_ (99.9% metals basis) into acetonitrile/ethylene glycol (35 vol.%/65 vol.%) in an N_2_ environment. The solutions were stirred for at least 2 h before use. All precursors and solvents were purchased from Sigma–Aldrich (Yongin-Si, Gyeonggi-do, Korea). The precursor solution concentration of HfO_2_ was 0.2 M. The concentration of the SnO_x_ precursor solution was 0.167 M, if not otherwise mentioned.

### 2.2. Thin-Film Transistor Fabrication and Characterization

Molybdenum was deposited by sputtering and patterned as the gate on a glass substrate. The HfO_2_ was fabricated by spin-coating the HfO_2_ precursor solution. The solution was first spin-coated at 2000 rpm during 25 s. Then, curing was performed during 3 min at 250 °C on a hotplate followed by UV treatment (320 W, wavelengths of 185 and 254 nm) at 100 °C. Spin-coating, curing, and UV treatment were repeated 4 times. The sample was then annealed at 350 °C for 2 h in air.

The SnO_x_ precursor solution was then spin-coated at 4000 rpm during 25 s. A first curing step at 100 °C was then followed by a second curing step at 280 °C. Each curing step lasted for 5 min. 

Then, an active island was made. After a 2 h annealing step at 300 °C, via-holes were made to reach the gate contacts. Finally, indium zinc oxide (10 wt % In_2_O_3_) was deposited by sputtering and patterned as the source/drain. The samples had a final annealing step at 300 °C for 2 h in air on a hotplate.

The TFT IV curves were measured with a 4156B semiconductor parameter analyzer (HEWLETT PACKARD, Palo Alto, CA, USA). Twenty-five TFTs with a width (W) of 50 µm and a length (L) of 10 µm were measured to obtain average and standard deviation values. For simplicity, the threshold voltage was determined from the value at which the TFT reached I_DS_ = W/L × 10^−10^ A at a drain voltage V_DS_ = 0.1 V. The saturation mobility was extracted from the linear part of the √I_DS_ versus V_GS_ curve. The I_On_/I_Off_ ratio was extracted at V_DS_ = 1 V from the ratio of maximum I_DS_ and minimum I_DS_. The C-f characteristics of HfO_2_ were measured using a MIM structure (Mo/HfO_2_/Mo) with an Agilent E4980A precision LCR meter (Agilent Technologies, Inc., Santa Clara, CA, USA).

### 2.3. Thin Film Characterization

For atomic force microscopy (AFM) and X-ray spectroscopy (XPS), thin films were characterized with the same condition used in TFTs. For X-ray diffraction (XRD), X-ray reflectivity (XRR), and Hall effect, a 40 nm thick a-SnO_x_ (using a 0.2 M precursor solution, coating and curing at 100 °C and 280 °C twice before annealing at 300 °C) was used for characterization. Atomic force microscopy was measured using an XE-100 (Park Systems, Suwon-Si, Gyeonggi-do, Korea) in tapping mode. Both XRD and XRR data were acquired with Cu-Kα radiation (λ = 1.54 Å). Time of flight secondary ion mass spectroscopy (TOF-SIMS) was measured using a TOF.SIMS 5 (IONTOF, Münster, Germany). The Cs^+^ ions were used as the sputtering ions at 1 keV, with a current of 10 nA.

The XPS was performed using a PHI 5000 VersaProbe (Ulvac-PHI, Kanagawa, Japan) at a base pressure of 2.0 × 10^−7^ Pa by calibrating with the C1s peak at 284.6 eV. The a-SnO_x_ was deposited on the HfO_2_ for analysis.

The HR-TEM measurements were performed using an Titan 80–300 (Thermo Scientific™, former FEI, Hillsboro, OR, USA) on a TFT cut using the Focused Ion Beam (FIB) technique with a Nova 600 NanoLab (Thermo Scientific™, Former FEI, Hillsboro, OR, USA). A thin carbon layer was deposited to protect the sample from damage during FIB sampling. The Hall effect was measured with an ECOPIA HMS-3000 Hall effect measurement system (Ecopia, Anyang-si, Gyeonggi-do, Korea) using a Van der Pauw configuration. The average over 15 points were used to evaluate the Hall coefficient.

## 3. Results and Discussion

**Figure 1:**
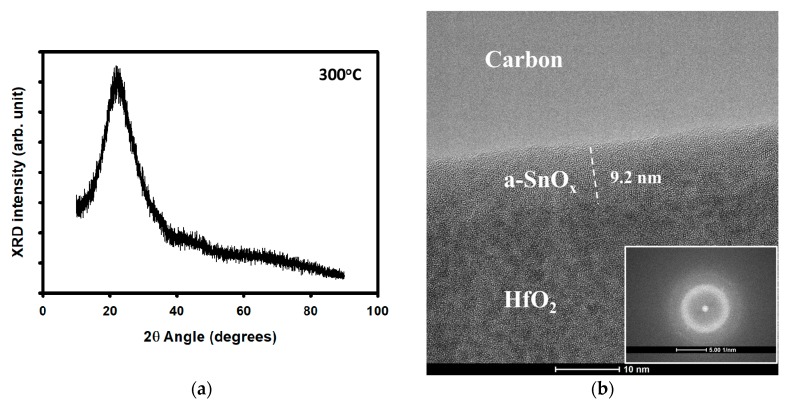

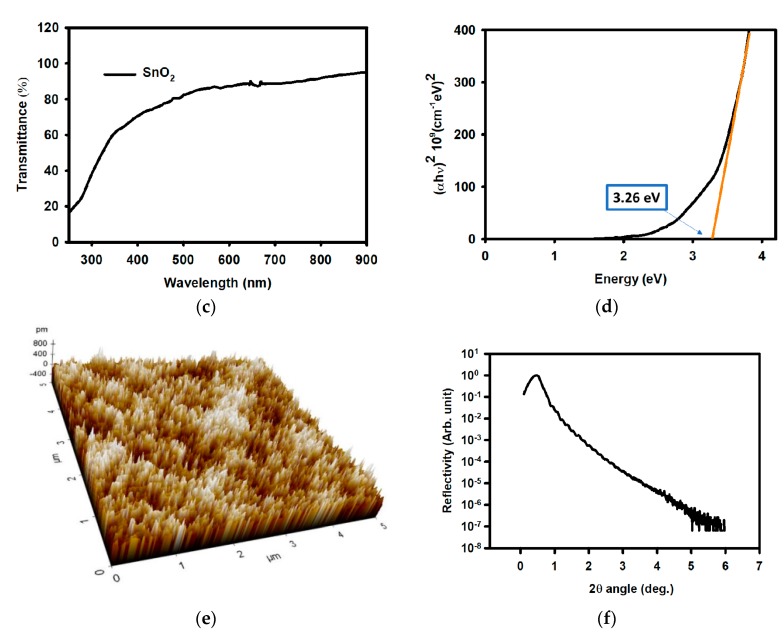
Physical properties of a-SnO_x_ thin films annealed at 300 °C. (**a**) XRD pattern of a 40 nm thick SnO_x_. (**b**) High-resolution TEM image of SnO_x_ used in a thin-film transistor (TFT) channel region; the inset shows the local fast Fourier transform (FFT) pattern. (**c**) Transmittance of a-SnO_x_ thin film and (**d**) extraction of a-SnO_x_ optical bandgap using a Tauc plot. The orange line is the extrapolation from the curve and the intersection with the abscissa axis gives the optical bandgap; the value is indicated in the blue box. (**e**) An AFM image of the a-SnO_x_ surface. (**f**) The XRR measurement of the a-SnO_x_. In (**a**,**f**) arb. Stands for arbitrary.

Figure 1 shows the thin film characteristics of solution processed tin oxide annealed in air at 300 °C. Figure 1a,b depicts the crystallinity as measured by a 2500D XRD system (Rigaku, Tokyo, Japan) and HR-TEM, respectively. The XRD pattern shows the broad amorphous peak related to the glass substrate [17]. The HR-TEM and the inset—showing the local fast Fourier transform (FFT)—further demonstrate the amorphous phase of SnO_x_.

Figure 1c,d shows the optical characteristics of the a-SnO_x_, which had a transmittance of 85.9% at 550 nm. The optical bandgap was extracted from the (αhυ)^2^ versus photon energy, as shown in Figure 1d [21]. The optical bandgap was 3.26 eV. The value was smaller than the polycrystalline counterpart (3.6 eV) [22] and is further discussed in this paper.

The surface roughness was analyzed via AFM, and the results are shown in Figure 1e. The root mean square (RMS) roughness was 0.155 nm. The density of the material was evaluated by XRR (Figure 1f) and showed a density of 5.29 g/cm^3^. The value was smaller than the density of polycrystalline SnO_2_, but similar to the one reported by Huang et al. [17] for the amorphous phase of SnO_2_ (5.01 g/cm^3^). The density and the optical bandgap of the a-SnO_x_ were smaller than the values of crystalline SnO_2_. The reason could be that denser metal oxide materials have a higher density of overlap of the s-orbitals which, in turn, leads to a higher bandgap [23].

**Figure 2:**
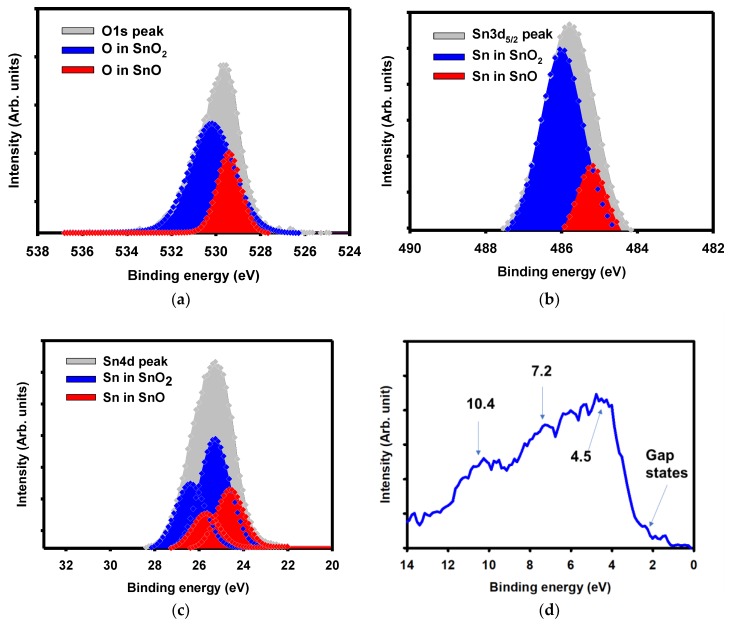

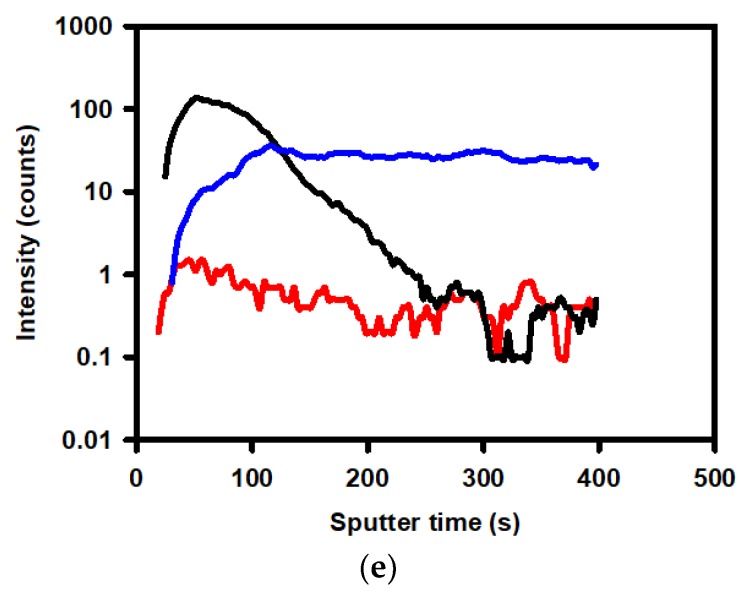
Composition analysis of a-SnO_x_. (**a**) O1s spectrum and decomposed components of a-SnO_x_ thin film. The blue part represents oxygen in SnO_2_, the red one oxygen in SnO. (**b**) Deconvolution of the Sn3d 5/2 peak. The blue part represents Sn as in SnO_2_, the red part Sn as in SnO. (**c**) Sn4d peak analysis. The two doublets related to SnO_2_ are in blue, the doublets related to SnO are in red. (**d**) Valence band analysis of a-SnO_x_. (**e**) TOF-SIMS profile of a-SnO_x_. In blue, red, and black are shown CsSi+, CsC+, and CsSn+ depth profile, respectively. In the above charts, Arb. stands for arbitrary.

The electronic configuration of a-SnO_x_ is shown in Figure 2. The O1s, Sn 3d5/2, Sn4d, and the valence band as measured by XPS are shown in Figure 2a–d. The deconvolution of the O1s follows the analysis used for tin oxide and not the conventional metal-oxide analysis [24,25,26]. The O1s peak in metal-oxides compounds are conventionally analyzed as having three parts: metal-oxygen, oxygen vacancies, and –OH groups. Here, the deconvolution of the O1s peak led to the knowledge of the phase composition of the film. The O1s can be deconvoluted into two peaks centered at 530.1 eV and at 529.4 eV. The separation of the two peaks by 0.7 eV indicates that the former was related to oxygen as in SnO_2_ while the latter was related to SnO [23,24]. The relative ratios of each component led to the presence of 72% of SnO_2_ and 28% of SnO. The O:Sn ratio was therefore 1.72. The deconvolution of the O1s peak into only two peaks is further justified by the analysis of the Sn3d5/2 peak.

The Sn3d5/2 peak can be deconvoluted into two peaks as well: one at 485.2 eV and another one at 485.9 eV. The energy separation of 0.7 eV is understood as before as the presence of Sn^4+^ and Sn^2+^ species. The relative ratios led to a phase ratio with a dominance of SnO_2_ over SnO—77% and 23%, respectively; the ratio O:Sn was 1.77 and in concordance with the one presented for O1s.

The analysis of the Sn4d core level further confirmed the presence of SnO_2_ and SnO, as shown in Figure 2c. The spin-orbit doublet was separated by 1.1 eV and an intensity ratio of 3:2 fit both Sn^4+^ (in blue in Figure 2c) and Sn^2+^ (in red in Figure 2c) related doublets. Note that both parts of a doublet have the same full width at half maximum (FWHM). The doublet related to Sn^4+^ was positioned at 25.3 and 26.4 eV and the doublet for Sn^2+^ was positioned at 24.6 and 25.7 eV.

The presence of the SnO_2_ phase over the SnO phase suggests that the material was n-type. To confirm, Hall effect measurements were performed. A negative Hall coefficient of −2.7266 cm^3^/C confirmed the n-type character of the a-SnO_x_.

Figure 2d shows the valence band (VB) of the a-SnO_x_ as measured by XPS. The VB region usually shows three main peaks at 4.5, 7.2, and 10.4 eV [24]. The oxygen 2p bonding and anti-bonding related peak was at approximately 4.5 eV. The states resulting from the ppσ hybridization between Sn 5p and O 2p orbitals appeared at 7.2 eV, and bonds between the 5s and O2p orbitals appeared at 10.4 eV. Last but not least, the distance between the main peak (at 4.5 eV) and the 4d peak was ~21 eV and further confirmed the formation of SnO_2_ [26]. 

We evaluated the presence of carbon in the thin film by TOF-SIMS. We chose TOF-SIMS to evaluate the carbon content as the measurement gives higher precision than XPS. The depth profile of a-SnO_x_ is shown in Figure 2e; only traces of carbon were observed in the film. The result exhibits the quality of the process, leading to an amorphous tin oxide thin film without carbon contamination.

**Figure 3:**
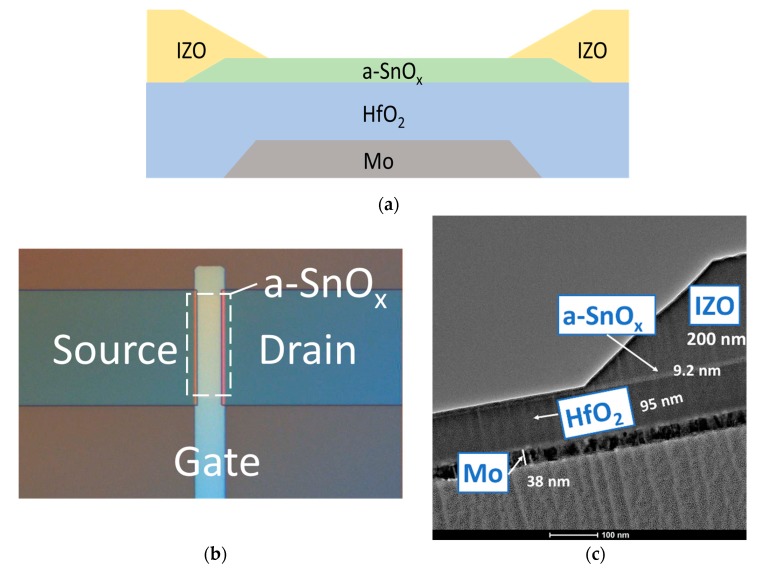
The a-SnO_x_ TFT structure: (**a**) a schematic diagram of the bottom gate and top contact a-SnO_x_ TFT. (**b**) An illustration of an a-SnO_x_ TFT. (**c**) TEM image of an a-SnO_x_ TFT taken near the source/drain region.

For potential use of a-SnO_x_ in further applications, we employed a-SnO_x_ as the active material in TFTs. Figure 3a shows the structure of the TFT used. Figure 3b shows a top view of a a-SnO_x_ TFT taken by an optical microscope. Figure 3c shows a TEM image near the source/drain region. The thicknesses of Mo, HfO_2_, SnO_2_, and IZO were 38 nm, 95 nm, 9.2 nm, and 200 nm, respectively. 

**Figure 4:**
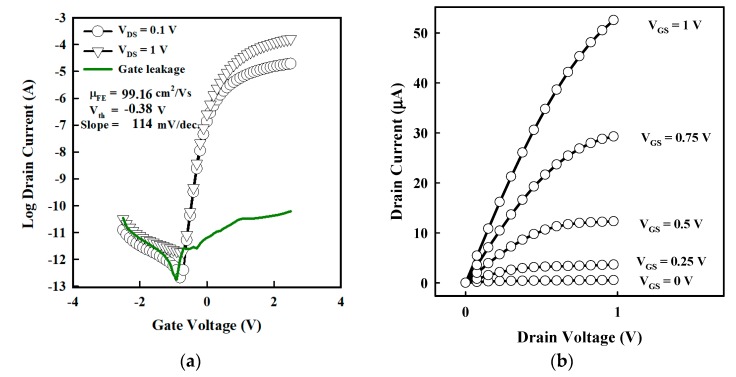
Electrical characterization of a-SnO_x_ TFT. Typical (**a**) transfer and (**b**) output of a TFT using HfO_2_ as the gate insulator. For the transfer curve, V_DS_ = 0.1 and 1 V are shown in dark lines with symbols. The green line is the gate leakage. For the output curves, V_DS_ was varied from 0 to 1 V, and V_GS_ was set from 0–1 V.

The TFTs were evaluated under DC operation; therefore, a low-frequency operation was used to evaluate the dielectric capacitance value. At 100 Hz, the capacitance of the HfO_2_ had a value of 219 nF/cm^2^. Note that the permittivity of HfO_2_ was 23, close to its theoretical value [27]. The TFTs field-effect mobility µ_FE_, threshold voltage (V_th_), subthreshold swing (S.S.), and I_ON_/I_OFF_ were 99.5 ± 15.4 cm^2^/Vs, −0.4 ± 0.04 V, 103 ± 16 mV/dec, and 2.2 ± 1.4 10^8^, respectively. The typical TFT transfer and output characteristics are shown in Figure 4a,b. The TFTs had µ_FE_, V_th_, SS, and I_ON_/I_OFF_ values of 99.16 cm^2^/Vs, −0.38 V, 114 mV/dec, and 1.7 × 10^8^. Note that the gate leakage was at least 10^5^ smaller than the I_DS_ current, ensuring that the TFT operation was not influenced by the gate leakage. 

**Figure 5:**
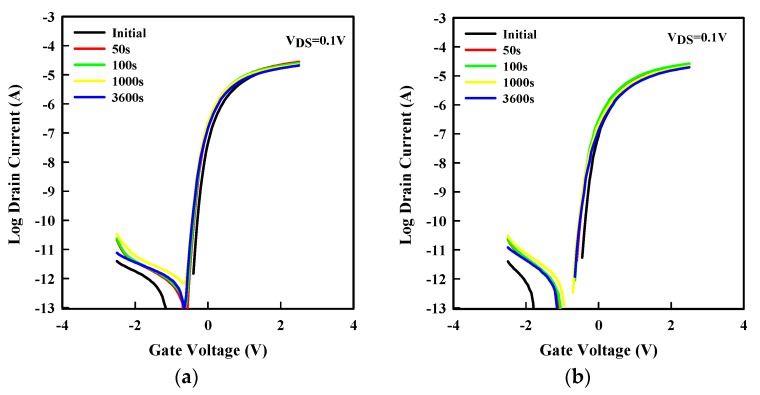
a-SnOx TFTs under various stress during 3600 s. The evolution of the transfer curve of a-SnO_x_ TFT under (**a**) positive gate bias stress and (**b**) negative bias stress. After 50, 100, 1000, and 3600 s, a transfer curve was measured (at V_DS_ = 0.1 V). One color is shown per measurement.

To evaluate the potential use of a-SnO_x_ in TFTs, other metrics such as stability under gate bias stress can be used. Positive and negative gate bias stresses were applied for 1 h under V_GS_ = 2.5 V and V_GS_ = −2.5 V, respectively. Figure 5a,b shows the evolution of the transfer curve under PBS and NBS, which led to a |ΔV_th_| of 0.15 V and 0.05 V, respectively. Note that most of the ΔV_th_ occurred within the first 50 s. It is suggested that defects are created near/at the dielectric interface with the a-SnO_x_ layer [4,10,12]. Also note an increase in the I_OFF_ during the applied stress. The increase in the OFF region can be understood as that more holes are available in the channel region. The holes may come from the created defects. Nonetheless, the I_OFF_ remains in the 10 pA range suggesting that the number of holes remained small. Higher stability may be achieved via surface treatment such as UV treatment or plasma treatment [28,29]. 

## 4. Conclusions

The properties of amorphous SnO_x_ were investigated. The amorphous phase was confirmed by both XRD and HR-TEM. The a-SnO_x_ was an n-type semiconductor with SnO_2_ as the main phase; the material had a density of 5.29 g/cm^3^; it showed a transmittance of 85.9% at 550 nm; and the a-SnO_x_ demonstrated a band gap of 3.26 eV. Implementation in TFTs with HfO_2_ as the dielectric led to field-effect mobilities of 99.5 cm^2^/Vs and I_ON_/I_OFF_ over 10^7^ at operating voltages below 3 V. 

The proposed solution processed a-SnO_x_ was an amorphous metal oxide semiconductor based on one cation only. The results demonstrated possible competition with other high-performance materials—including poly-Si and multiple cation-based AOS such as IGZO—and could potentially be used in a wider range of applications.
